# Endometriosis and Cardiovascular Disease: Exploring Pathophysiological Interconnections and Risk Mechanisms

**DOI:** 10.3390/diagnostics15121458

**Published:** 2025-06-08

**Authors:** Gabriela Szpila, Julia Szczotka, Alexander Suchodolski, Mariola Szulik

**Affiliations:** 1 4. Military Clinical Hospital with Polyclinic SP ZOZ in Wroclaw, 53-114 Wroclaw, Poland; gabrielaszpila99@gmail.com (G.S.); j.szczotkaa@gmail.com (J.S.); 2 Doctoral School, Medical University of Silesia in Katowice, 40-055 Katowice, Poland; alex.suchodolski@gmail.com; 3 Department of Cardiology and Electrotherapy, Silesian Center for Heart Diseases, Faculty of Medical Sciences in Zabrze, Medical University of Silesia in Katowice, 40-055 Katowice, Poland; 4 Collegium Medicum—Faculty of Medicine and Faculty of Applied Sciences, Department of Medical and Health Sciences, WSB University, 41-300 Dąbrowa Górnicza, Poland

**Keywords:** endometriosis, cardiovascular disease, atherosclerosis, inflammation

## Abstract

Endometriosis, traditionally viewed as a gynecological disorder, is increasingly recognized as a systemic disease with significant cardiovascular implications. Recent studies suggest that women with endometriosis are at higher risk for developing atherosclerosis and other cardiovascular diseases (CVDs), due to chronic systemic inflammation, endothelial dysfunction, oxidative stress, and metabolic disturbances. This review aimed to summarize current evidence on the vascular implications of endometriosis. A literature search was conducted in PubMed and Google Scholar, focusing on studies exploring the relationship between endometriosis and cardiovascular risk. In rare cases, endometriosis can affect extrapelvic locations such as the diaphragm or pericardium, presenting with cyclical chest pain or dyspnea and mimicking cardiopulmonary conditions. These atypical manifestations often delay diagnosis and highlight the need for heightened clinical awareness. Advances in imaging and minimally invasive techniques, including robotic surgery, have improved the detection and management of such presentations. Shared molecular pathways between endometriosis and CVDs, including pro-inflammatory cytokines and metabolic dysregulation, provide a rationale for exploring novel therapeutic approaches. Emerging pharmacologic options such as statins, metformin, or antiplatelet agents may offer dual benefits for both reproductive and cardiovascular health. Given the multifactorial nature of endometriosis, a multidisciplinary approach involving gynecologists, cardiologists, and primary care providers is essential. These findings highlight the need for early cardiovascular risk assessment and tailored preventive strategies in this population.

## 1. Introduction

Endometriosis is a chronic condition characterized by the presence of tissue similar to the endometrium (the uterine lining) growing outside the uterus. This abnormal tissue often affects the ovaries, fallopian tubes, and pelvic structures but can occasionally extend to distant organs [1]. Globally, according to the World Health Organization (WHO), approximately 10% of women of reproductive age—equivalent to nearly 190 million individuals—are affected by endometriosis [2].

Despite its prevalence, the precise etiology of endometriosis remains elusive, with multiple hypotheses proposed to explain its development. The theory of retrograde menstruation, one of the most widely discussed, suggests that menstrual blood containing endometrial cells flows backward through the fallopian tubes into the peritoneal cavity, where these cells implant and proliferate. Another explanation, peritoneal metaplasia, posits that peritoneal cells transform into endometrial-like cells due to environmental or genetic triggers. Immune dysfunction is also implicated, as it may allow endometrial cells to survive and thrive in ectopic locations by evading immune surveillance mechanisms. Genetic predisposition plays a role as well, with endometriosis more frequently observed in women with a familial history of the disease. Research has also identified genetic variants associated with an increased risk of endometriosis. Furthermore, the cellular dissemination hypothesis proposes that endometrial cells spread via blood or lymphatic pathways, accounting for rare cases of extra-abdominal endometriosis [3,4].

The clinical manifestations of endometriosis are diverse, with hallmark symptoms including dysmenorrhea (severe menstrual pain), dyspareunia (pain during intercourse), and chronic pelvic pain. Other symptoms may include bowel or urinary discomfort, heavy menstrual bleeding, intermenstrual spotting, infertility, fatigue, and gastrointestinal disturbances such as bloating, nausea, diarrhea, or constipation [1,4]. These symptoms can overlap with conditions like irritable bowel syndrome, complicating diagnosis [5].

Inflammatory processes associated with endometriosis lead to elevated levels of pro-inflammatory cytokines, such as IL-6 and TNF-α, as well as increased oxidative stress. These same mediators play a pivotal role in the pathogenesis of atherosclerosis, contributing to endothelial dysfunction and the formation of atherosclerotic plaques. Moreover, immunological disturbances present in endometriosis may exacerbate inflammatory processes within blood vessels, increasing the risk of developing atherosclerosis [6,7].

This review aims to consolidate current knowledge on cardiovascular factors and pathogenesis, clinical presentation, diagnostic approaches, and therapeutic options for endometriosis.

## 2. Materials and Methods

This review is based on a literature search conducted in PubMed and Google Scholar, focusing on studies published between 2010 and 2024 related to endometriosis and cardiovascular risk. Keywords included “endometriosis,” “cardiovascular disease,” “atherosclerosis,” and related terms. Priority was given to original research, systematic reviews, and meta-analyses.

To ensure methodological rigor and relevance, the inclusion criteria were as follows:

- Original studies, systematic reviews, or meta-analyses published in peer-reviewed journals within the last 15 years.
- Human studies explicitly investigating the association between endometriosis and cardiovascular outcomes, including atherosclerosis, endothelial dysfunction, oxidative stress, and metabolic alterations.
- Articles available in English and providing sufficient methodological detail.

Exclusion criteria included the following:

- Publications limited to in vitro or animal model studies without clinical translation.
- Non-peer-reviewed materials such as editorials, commentaries, conference abstracts, and opinion pieces.
- Studies unrelated to cardiovascular outcomes or lacking in relevance to the pathophysiological mechanisms discussed in this review.

No new data were generated, and no ethical approval was required. Generative artificial intelligence tools were used to assist in identifying relevant articles and refining search strategies.

## 3. Mechanisms Linking Endometriosis and Cardiovascular Disease

### 3.1. Associations Between Endometriosis and Cardiovascular Risk Factors

Oxidative stress contributes to both endometriosis and cardiovascular risk by generating reactive oxygen species (ROS) that lead to chronic inflammation, cellular damage, and tissue changes. In endometriosis, oxidative stress damages local tissues, promotes inflammation, and facilitates lesion growth by affecting immune responses, particularly through the activation of macrophages and other immune cells. This inflammation and oxidative environment support ectopic endometrial tissue’s survival and growth, worsening endometriosis symptoms, and potentially harming ovarian and reproductive function [8,9].

The same mechanisms of oxidative stress that exacerbate endometriosis also contribute to CVD by damaging endothelial cells, impairing vascular function, and increasing atherosclerosis risk. The chronic inflammatory environment driven by oxidative stress and immune cell activation is a known contributor to vascular damage, which increases susceptibility to CVD. Research indicates that women with endometriosis exhibit increased levels of oxidative stress markers, including reactive oxygen species (ROS) and oxidative damage byproducts such as malondialdehyde (MDA). In addition, alterations in the activity of key antioxidant enzymes, such as superoxide dismutase (SOD) and catalase (CAT), have been observed, reflecting an imbalance between oxidative stress and antioxidant defenses. While these changes contribute to the pathogenesis and progression of endometriosis, they may also increase susceptibility to cardiovascular disease by promoting endothelial dysfunction and vascular inflammation. Nuclear factor kappa B (NF-κB), a transcription factor activated in response to oxidative stress, plays a central role in amplifying inflammatory signaling pathways rather than serving as a direct oxidative stress marker [8,9].

One study has focused on oxidative stress biomarkers and the subsequent impact on cardiovascular risk in endometriosis patients, such as elevated levels of pro-inflammatory cytokines like thymic stromal lymphopoietin (TSLP), TNF-α, and IL-1β. Oxidative markers like malondialdehyde (MDA) were observed, supporting the link between endometriosis-associated oxidative stress and CVD risk factors. Additionally, the findings suggest that oxidative stress in endometriosis patients may impair normal immune responses, intensifying inflammation that further affects cardiovascular health [10].

Studies show that endometriosis significantly impacts endothelial health, which can lead to arterial stiffness and endothelial dysfunction, both known factors in CVD development. Research highlights that women with endometriosis often exhibit elevated levels of arterial stiffness and reduced endothelial function, which increase their CVD risk. For instance, studies using non-invasive Endo-PAT measurements have found lower Reactive Hyperemia Index (RHI) values in endometriosis patients, a marker of endothelial dysfunction, compared to controls. This dysfunction, along with greater arterial stiffness measured by the Augmentation Index (AI), signals a higher cardiovascular risk among these patients [11,12].

**Figure 1:**
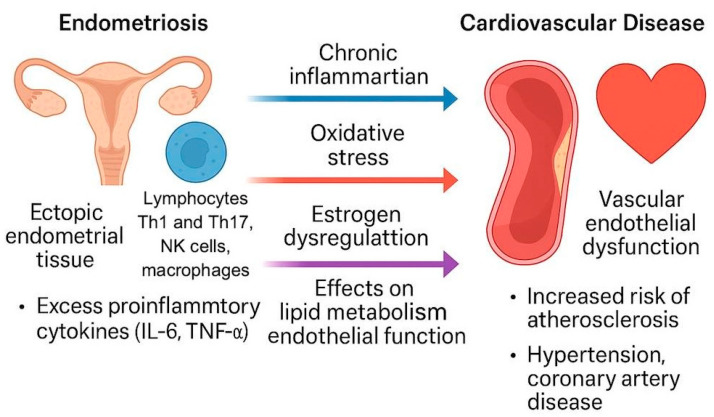
Mechanisms linking endometriosis with cardiovascular disease.

Estrogen plays a complex role in both endometriosis and CVD, primarily through its effects on vascular condition, oxidative stress, and inflammation. In women, estrogen protects cardiovascular safety by maintaining endothelial protective effects via estrogen receptor action, such as Erα, ERβ, and GPR30, reducing oxidative stress and influencing the lipid profile. Estrogen achieves this as a membrane-associated receptor. Through these receptors, estrogen promotes vasodilation, modulates nitric oxide pathways, and limits vascular inflammation, reducing the risk of endothelial dysfunction, arterial stiffness, and atherosclerosis. These benefits are diminished with lower estrogen levels in postmenopausal women, contributing to a higher CVD risk (Figure 1).

Additionally, studies highlight estrogen’s protective effects through antioxidant pathways, where it regulates oxidative stress markers, such as superoxide dismutase (SOD2). This antioxidant action mitigates the oxidative damage often exacerbated in cardiovascular and metabolic diseases, a pathway also relevant in endometriosis, where increased oxidative stress and inflammation drive disease progression [13,14].

### 3.2. Endometriosis and Atherosclerosis Shared Pathophysiological Mechanisms (Figure 1)

Emerging research indicates a potential association between endometriosis and cardiovascular disease (CVD), with some studies suggesting an increase in arterial stiffness and endothelial dysfunction in individuals affected by endometriosis. These vascular changes may contribute to a pro-atherogenic environment; however, the extent to which they directly influence atherosclerotic processes remains under investigation.

Immune cells play a crucial role in the pathophysiology of endometriosis. Ectopic endometrial lesions are infiltrated by macrophages secreting pro-inflammatory cytokines such as interleukin-6 (IL-6) and tumor necrosis factor-alpha (TNF-α), sustaining chronic local inflammation. Additionally, T lymphocytes, particularly Th1 and Th17 subsets, exacerbate this inflammatory milieu and may disrupt immune tolerance. Natural killer (NK) cells show reduced cytotoxic activity, facilitating the survival and proliferation of ectopic tissue. This localized immune dysregulation may influence endothelial function and contribute to subtle vascular remodeling observed in endometriosis [1].

The chronic inflammatory milieu and oxidative stress associated with endometriosis may modulate endothelial function and promote subtle vascular remodeling. Nonetheless, it is important to note that the inflammatory response in endometriosis is primarily localized to the pelvic cavity, and evidence supporting a systemic inflammatory effect sufficient to drive atherogenesis is currently limited [15,16].

Additionally, while endometriosis-associated inflammation may theoretically enhance the recruitment of immune cells, the formation of foam cells—a hallmark of atherosclerotic plaque—primarily arises from the uptake of modified lipoproteins by macrophages within the arterial intima. This process is driven by systemic lipid abnormalities and chronic vascular inflammation, mechanisms not directly linked to endometriosis itself. Thus, while endometriosis may contribute to a pro-inflammatory systemic environment, its role in directly accelerating atherosclerotic plaque formation requires further elucidation [15,16].

These observations highlight the importance of assessing cardiovascular risk profiles in patients with endometriosis, particularly given the potential additive effect of shared risk factors such as dyslipidemia, insulin resistance, and chronic inflammation. Early detection and management of cardiovascular risk factors may play a crucial role in reducing the long-term burden of atherosclerotic disease in this population [15,16].

### 3.3. Genetic Links Between Cardiovascular Disease and Endometriosis

Recent genome-wide association studies (GWAS) have highlighted genetic loci, notably CDKN2B-AS1 on chromosome 9p21 and 7q22, as overlapping risk factors for both endometriosis and CVD. The CDKN2B-AS1 locus is notably associated with conditions like abdominal aortic aneurysm and ischemic stroke, suggesting its crucial role in vascular health by affecting inflammatory and cell-proliferation pathways common to both CVD and endometriosis [17]. Additionally, studies have linked single-nucleotide polymorphisms (SNPs) in CDKN2B-AS1 with the severity of atherosclerosis, while 7q22 is also associated with genetic susceptibility to both conditions [18]. These genetic overlaps suggest a common susceptibility to chronic inflammation, a key feature in both CVD and endometriosis. The genetic markers are thought to influence shared mechanisms like immune response, cellular adhesion, and angiogenesis, pathways that can exacerbate both cardiovascular conditions and endometriotic tissue proliferation [17,18].

### 3.4. Association of Endometriosis with Atherosclerosis and Its Complications

Chronic inflammation, excessive oxidative stress, and hormonal imbalances are shared biological mechanisms underlying both endometriosis and CVD. These overlapping pathways may explain the strong association between the conditions, as increasingly highlighted in scientific research. Women with endometriosis frequently exhibit elevated levels of inflammatory markers such as CRP, fibrinogen, homocysteine, and interleukins, which can amplify pathological processes in the vascular walls, thereby raising the risk of atherosclerosis, ischemic stroke, and myocardial infarction. This emerging evidence underscores the need for the heightened awareness of cardiovascular risks in women with endometriosis and encourages further investigation into targeted prevention and intervention strategies for this population [19,20].

This phenomenon is not confined solely to inflammatory markers. Clinical observations suggest that women with endometriosis also experience lipid abnormalities and hypertension, both of which significantly amplify the risk of cardiovascular disease. Excess estrogen in endometriosis affects lipid metabolism by increasing LDL levels, while hormonal imbalances may enhance the activity of the renin–angiotensin–aldosterone system, leading to elevated blood pressure. Hormonal treatments, such as progestins and estrogens, can further worsen the lipid profile and raise blood pressure in susceptible individuals.

Endometriosis is also associated with metabolic syndrome, which includes insulin resistance, dyslipidemia, and obesity. Insulin resistance exacerbates lipid disorders and contributes to hypertension through sympathetic nervous system activation and sodium retention in the kidneys [21,22,23,24].

Furthermore, endothelial dysfunction—critical for regulating blood flow—has been observed in these patients, leading to thrombosis and further increasing the likelihood of major cardiovascular events. This underscores the need for a comprehensive approach to risk assessment and management in women with endometriosis, focusing on both systemic inflammation and vascular health [15].

The findings from the Nurses’ Health Study II, which included thousands of women with laparoscopically confirmed endometriosis, revealed a striking 62% higher risk of coronary heart disease compared to a control group. In the article written by K. Okoth et al., similar findings were reported in a retrospective analysis carried out in the United Kingdom. The analysis revealed that women with endometriosis have a 40% higher likelihood of developing ischemic heart disease compared to those without this diagnosis. Furthermore, the study indicated that endometriosis may be associated with a 26% increased risk of arrhythmia [25]. This emphasizes the critical need for targeted preventive measures in this population. Additionally, increased arterial stiffness, often observed in young women with endometriosis, suggests that early interventions could play a pivotal role in reducing the risk of atherosclerosis and related complications.

These alarming statistics underscore the importance of further research into the intricate interplay between endometriosis and cardiovascular health to develop more effective strategies to protect women affected by this condition [20,26,27].

### 3.5. Supradiaphragmatic Endometriosis: Clinical Cases and Diagnostic Challenges

Supradiaphragmatic endometriosis, involving the diaphragm, pleura, and pericardium, represents a rare and often misdiagnosed variant of extrapelvic endometriosis. This condition poses significant diagnostic challenges, as it frequently mimics symptoms typically associated with cardiopulmonary diseases. Clinical manifestations may include cyclic chest pain, dyspnea, and even recurrent pneumothorax [28,29,30]. Patients may also experience chest pain, shortness of breath, palpitations, fatigue, or fainting, with symptoms often worsening during menstruation. The exact mechanism remains unclear, but retrograde menstruation is a key theory. This process involves menstrual debris entering the pelvic cavity and spreading to the pericardium via the bloodstream or lymphatic system [31,32,33,34,35].

Pericardial endometriosis, a rare manifestation of endometriosis, occurs when endometrial-like tissue implants within the pericardium, potentially leading to significant cardiac symptoms. One of the most common cardiac symptoms is pericardial effusion—an abnormal accumulation of fluid in the pericardial sac surrounding the heart. On echocardiography, this manifests as an anechoic space surrounding the heart [32,33].

Due to the nonspecific nature of these symptoms and their overlap with cardiovascular pathologies, accurate and timely diagnosis is frequently delayed. The following clinical cases illustrate the variability in presentation, complexity of diagnosis, and necessity of multidisciplinary treatment strategies.

One illustrative case, described by Takigawa et al., involved a 46-year-old woman presenting with recurrent pneumothorax, closely associated with her menstrual cycle. This condition, known as catamenial pneumothorax, is a classical but rare presentation of thoracic endometriosis. During surgical intervention, a small lesion resembling a dark, berry-like spot was identified on the pericardium. Histopathological analysis of the resected tissue revealed the presence of ectopic endometrial tissue, confirmed by immunohistochemical staining positive for estrogen and progesterone receptors. This finding supported the diagnosis of pericardial endometriosis. Postoperative hormonal therapy led to the complete resolution of the patient’s symptoms, with no recurrence observed during follow-up. This case exemplifies how hormonally active ectopic endometrial tissue can be responsible for cyclical thoracic symptoms and how surgical confirmation combined with hormonal suppression can yield favorable outcomes [31].

Another case, reported by Ceccaroni and collaborators, involved a patient diagnosed with extensive endometriosis affecting multiple anatomical compartments, including the pericardium, pleura, diaphragm, pelvic peritoneum, and bowel. Diaphragmatic lesions were primarily unilateral, consistent with prevailing theories suggesting that retrograde menstruation and the directional flow of peritoneal fluid contribute to the right-sided predilection of thoracic endometriosis. The patient presented with vague thoracoabdominal discomfort, and preoperative imaging did not clearly reveal the extent of thoracic involvement. Surgical management required a multidisciplinary approach tailored to the patient’s age, fertility goals, and the severity and distribution of lesions. This case highlights the importance of considering a patient’s reproductive future and individualizing care plans in managing complex forms of extrapelvic endometriosis [36].

In a further example, Bachi et al. demonstrated the use of a combined robotic-assisted laparoscopic and thoracoscopic approach for the management of severe diaphragmatic, pleural, and pericardial endometriosis. The surgical team, composed of gynecologic and thoracic surgeons, successfully excised multiple lesions from all involved thoracic structures. This approach allowed for the precise identification and complete resection of the lesions with minimal invasiveness. The patient experienced significant symptomatic relief postoperatively, and no recurrences were observed at subsequent evaluations. This case underscores the effectiveness of multidisciplinary collaboration and the role of advanced surgical techniques in managing extensive thoracic endometriosis [37].

A similar case was described by Nguyen et al., where a 35-year-old woman experienced pain in the chest and right upper extremity, which was initially suspected to be of cardiac origin. Subsequent evaluations and intraoperative findings revealed full-thickness endometrial lesions involving the diaphragm and pericardium. These were removed using a robotic-assisted laparoscopic approach. Histological analysis confirmed the endometrial origin of the lesions. Following surgery, the patient’s symptoms resolved entirely. This case is emblematic of the potential for misdiagnosis due to symptom overlap with cardiovascular disease, and it highlights the utility of minimally invasive surgery in both diagnosis and treatment (Table 1) [38].

Across all these cases, a common thread emerges: the clinical presentation of supradiaphragmatic endometriosis often closely resembles that of cardiothoracic conditions, leading to diagnostic confusion and delays. Traditional diagnostic workups focused solely on cardiac or pulmonary causes may fail to identify the gynecologic etiology, especially when a cyclical pattern of symptoms is overlooked. This underlines the necessity for increased clinical awareness among non-gynecologic specialists, particularly cardiologists and pulmonologists, when evaluating reproductive-age women with unexplained thoracic symptoms.

In cases of suspected cardiac endometriosis, advanced diagnostic modalities such as magnetic resonance imaging (MRI) and echocardiography are utilized to detect abnormalities, including unusual masses or structural anomalies in the heart. These imaging techniques can also identify pericardial effusion and localized lesions that may indicate the presence of ectopic endometrial tissue. Confirmation of the diagnosis, however, requires histopathological analysis, typically obtained through biopsy or surgical exploration. Surgical excision of the affected tissue may be necessary when cardiac function is severely compromised, or if the patient experiences significant clinical symptoms. Although nuclear imaging techniques such as positron emission tomography (PET) and technetium-99m scintigraphy can provide valuable information about inflammatory and metabolic changes, these modalities are rarely employed in clinical practice due to their limited availability and specificity. Consequently, the diagnostic approach for cardiac endometriosis primarily relies on more conventional imaging methods, including echocardiography, computed tomography (CT), and MRI, which are generally more accessible and sufficient for identifying structural cardiac anomalies. It is important to note that endometrial deposits near the heart are exceptionally rare, underscoring the need for a high index of suspicion and careful diagnostic evaluation [32,39,40].

The effective treatment of endometriosis, especially when it involves thoracic or supradiaphragmatic regions, necessitates a comprehensive and interdisciplinary clinical approach. Hormonal therapies continue to form the foundation of management, particularly in scenarios where complete surgical excision is either impractical or contraindicated. Medications such as gonadotropin-releasing hormone (GnRH) agonists, progestogens, and combined oral contraceptives are commonly used to suppress endogenous estrogen production, thereby attenuating the proliferation and activity of ectopic endometrial lesions [41].

However, pharmacologic interventions alone are frequently inadequate when disease extends into the thoracic cavity, particularly affecting the pleura or pericardium. In these instances, surgical intervention becomes indispensable for both definitive diagnosis and therapeutic resolution. Innovations in minimally invasive surgery, such as video-assisted thoracoscopic surgery (VATS) and robotic-assisted laparoscopy, have considerably enhanced patient outcomes by enabling meticulous lesion excision with lower perioperative risk and reduced convalescence periods [42].

In addition to the physiological burden, the psychological and social impacts of undiagnosed thoracic endometriosis are significant. Affected individuals often present with symptoms that mimic cardiovascular conditions—such as chest discomfort, shortness of breath, and palpitations, leading to repeated emergency visits and unnecessary cardiac evaluations. This diagnostic ambiguity contributes to increased psychological distress and deteriorated quality of life. Timely and accurate identification of the disease, combined with personalized treatment plans, not only mitigates physical symptoms but also offers meaningful psychological benefits. These factors highlight the necessity of adopting a holistic, patient-oriented framework in clinical care [41,42].

To conclude, although supradiaphragmatic endometriosis is relatively uncommon, it should be included in the differential diagnosis of cyclic thoracic symptoms in women of reproductive age. Given its diverse clinical presentations, clinicians must maintain a high degree of suspicion, perform detailed history-taking with attention to menstrual patterns, and apply targeted imaging and surgical techniques. Prompt diagnosis and appropriate intervention can lead to markedly improved outcomes and can enhance patients’ overall quality of life.

## 4. Implications for Women’s Health and Future Research

While hormonal treatments and surgery remain the primary therapeutic options, recent studies have explored the potential benefits of various pharmacological agents. Among these, antiplatelet agents, beta-blockers, statins, and metformin have shown promise. Antiplatelet drugs, such as aspirin, may help reduce inflammation and limit the progression of endometrial lesions by inhibiting platelet aggregation and thromboxane production, with some evidence suggesting that endometriosis may have a prothrombotic component [43]. Beta-blockers, primarily used in cardiovascular conditions, have been investigated for their potential to alleviate pain and inflammation in endometriosis by modulating sympathetic nervous system activity and reducing pro-inflammatory cytokine release [44,45]. Statins, commonly used to manage cholesterol, possess anti-inflammatory properties that could reduce the severity of endometriosis-related pain and lesion progression by inhibiting inflammatory mediators like cytokines and prostaglandins [46]. Lastly, metformin, a medication typically prescribed for type 2 diabetes, may provide adjunctive benefits in endometriosis treatment by addressing insulin resistance and inflammation, as well as modulating pathways like mTOR and reducing the expression of vascular endothelial growth factor (VEGF), which plays a key role in lesion angiogenesis [47]. While these agents show considerable promise, further research is essential to better understand their full potential in treating endometriosis, especially for patients who are unresponsive to conventional therapies.

These findings have important implications for long-term health monitoring in women with endometriosis. Recognizing the association with CVD may lead to earlier and more targeted preventive measures, such as lifestyle interventions, more frequent cardiovascular screenings, and potentially new therapeutic approaches that address both reproductive and cardiovascular health. Future studies are expected to further clarify how endometriosis treatment can influence cardiovascular outcomes and identify which subgroups of patients may be at the highest risk.

However, there remains a significant research gap regarding the development of diagnostic tools that effectively assess cardiovascular risk specifically in women with endometriosis. Moreover, optimal treatment strategies that address both endometriosis and comorbid cardiovascular conditions are not yet well established, particularly concerning the long-term safety and efficacy of pharmacological agents like statins and metformin in this dual context.

Understanding this link between endometriosis and cardiovascular disease could ultimately lead to a more integrated approach to women’s health, acknowledging the intersection of reproductive and cardiovascular health.

## 5. Conclusions

In conclusion, endometriosis elevates the risk of atherosclerosis and other cardiovascular diseases through systemic inflammation, vascular dysfunction, and metabolic alterations. Future research should focus on clarifying these mechanisms and developing integrated care strategies to address both reproductive and cardiovascular health. Given the complex and multifactorial nature of endometriosis, a multidisciplinary approach involving gynecologists, cardiologists, and primary care providers is essential. Increased awareness and earlier screening for cardiovascular risk factors in women with endometriosis could lead to timely interventions and improved long-term outcomes. Moreover, emerging pharmacological therapies targeting inflammation, oxidative stress, and metabolic pathways hold promise and warrant further investigation. A deeper understanding of the shared pathophysiology between endometriosis and CVD may pave the way for innovative treatment strategies that not only protect reproductive function but also cardiovascular health throughout a woman’s life.

## Figures and Tables

**Table 1:** Summary of reported cases of thoracic endometriosis.

Author (Ref.)	Patient Description	Thoracic Involvement	Symptoms	Diagnosis Method	Management and Outcome
Takigawa et al. [32]	46-year-old woman with recurrent catamenial pneumothorax	Pericardium	Cyclical pneumothorax	Surgery + histopathology + IHC (ER/PR positive)	Surgical removal + hormonal therapy → Full symptom resolution, no recurrence
Ceccaroni et al. [37]	Extensive endometriosis in multiple sites including thorax	Right-sided diaphragm, pleura, pericardium	Thoracoabdominal discomfort	Surgical exploration	Multidisciplinary surgery tailored to fertility and severity → Successful outcome
Bachi et al. [38]	Severe thoracic endometriosis	Diaphragm, pleura, pericardium	Not specified	Robotic-assisted laparoscopy + thoracoscopy	Robotic-assisted laparoscopic and thoracoscopic surgery → Symptomatic relief, no relapse
Nguyen et al. [39]	35-year-old woman with chest and right arm pain	Diaphragm and pericardium	Chest and right upper extremity pain	Intraoperative findings + histological analysis	Robotic-assisted excision → Full symptom resolution

## Data Availability

No new data were created.
